# Sense of macro-level belonging supported university students’ life satisfaction amid global threats: evidence from Latvia’s first COVID-19 wave

**DOI:** 10.3389/fpsyg.2025.1677010

**Published:** 2025-12-18

**Authors:** Aleksandrs Kolesovs

**Affiliations:** Department of Psychology, University of Latvia, Riga, Latvia

**Keywords:** global threats, pandemics, satisfaction with life, sense of belonging, perceived stability, path analysis

## Abstract

Dealing with global threats challenges individual wellbeing. This study tested a path model, predicting satisfaction with life by the joint factor of perceived epidemiological, ecological, and economic threats via the perceived stability of and belonging to one’s country. Data were collected during the first wave of the COVID-19 pandemic in Latvia in 2020 from 317 university students aged 18–48 years (*M* = 23.65, *SD* = 5.98, 73% females, 60% employed). The measurements included satisfaction with life; relational and spatiotemporal components of the sense of belonging to the country; perceived stability of the country; and perceived impact of epidemics, climate change, natural disasters, and economic crises. There were no direct effects of perceived threats on the variables, except for a positive association with relational belonging. Both components of belonging positively predicted satisfaction with life, and the relational component transferred a minimal positive indirect effect of perceived threats. The perceived stability of Latvia correlated with both components of belonging and did not affect satisfaction with life. Demographic covariates did not substantially alter the model. In summary, the macro-level sense of belonging showed the strongest association with satisfaction with life, aligning with previous findings on the activation of social ties under threatening conditions.

## 1 Introduction

Global threats change individual life (Holman and Silver, 2005; Fung and Carstensen, 2006) and impact wellbeing (Handschuh et al., 2024; Kaniasty et al., 2024; Rogowska et al., 2021), having multiple effects in health and coping domains (Gori et al., 2020; Kagee et al., 2024). Studies on perceived global threats have reported relatively high sensitivity among Latvian people to global challenges (Chzhen, 2016) and emerging doubts about the country’s stability (Kolesovs and Kashirsky, 2014). The COVID-19 pandemic was no exception to this trend (Gruchoła and Sławek-Czochra, 2021; Kolesovs et al., 2021). Moreover, the perceived impact of the pandemic on Latvia was linked to the increasing impact of natural disasters and economic crises (Kolesovs et al., 2021). Observed changes provided an opportunity to test the relationship between perceived threats and characteristics of the country and societal involvement as potential buffers against aversive global events (Handschuh et al., 2024; Kaniasty et al., 2024). As reflections on a distal social system (e.g., Bronfenbrenner and Morris, 2006), perceived characteristics of the country have been less frequently examined in research, with an emphasis instead placed on proximal systems and individual characteristics (Eder et al., 2021; Rogowska et al., 2021). Considering the perceived macro-level characteristics as a “shell” in the globally changing world, it was possible to assess their links to individual subjective wellbeing as a “core” characteristic. Therefore, this study tested a path model, predicting satisfaction with life by perceived epidemiological, ecological, and economic threats via the sense of belonging to and the perceived stability of Latvia.

Researchers analyze perceived threats using two main approaches. The first approach considers a single source of threat, such as an epidemic (e.g., Liu et al., 2021), global climate problems (e.g., Steg, 2023), terrorist attacks (e.g., Holman and Silver, 2005), or war (e.g., Kimhi et al., 2023) as an acute crisis or highly significant global challenge. Separately, the second approach takes into account multiple sources of threats, groups them, and compares the relative significance of threats across levels of social systems, countries, and situations (Boehnke et al., 1998; Kahn et al., 2022; Kasapoğlu et al., 2009).

Previous studies in Latvia (Kolesovs and Ruza, 2019; Kolesovs et al., 2021) revealed that various global threats form a factor among perceived impacts on the country. In addition, the model of perceived impacts was relatively stable under changing conditions (Kolesovs et al., 2021). However, the significance of epidemics, natural disasters, and economic crises differed before and at the start of the COVID-19 pandemic. This change concurs with close associations among environmental, health, and economic threats (Kahn et al., 2022). In 2020, the pandemic allowed me to test the effects of this group of perceived threats on individual wellbeing as an important indicator of healthy functioning under the pressure of the global challenge (Handschuh et al., 2024; Kagee et al., 2024; Rogowska et al., 2021).

Ecological systems theory (Bronfenbrenner and Morris, 2006) and its application to situations of social change (Pinquart and Silbereisen, 2004) consider mediation of higher-level changes by lower levels of social systems. The perceived instability of Latvia in the face of global threats (e.g., Kolesovs and Kashirsky, 2014) led the study to focus on perceived macro-level characteristics of the country and evaluate their effects in dealing with pandemic-related threats. The perceived stability of the country was selected as a predictor of a more positive country’s future, which was established in a mixed-methods intercultural study (Kolesovs and Kashirsky, 2014). Perceived stability links to community resilience (Gilbar et al., 2022) and provides a more positive frame for individual socialization (Nurmi, 2004). An emphasis on societal characteristics also aligns with a continuous discussion on integrating individual and social levels of subjective wellbeing, which emphasizes links among wellbeing at country, community, and individual levels (Torres-Vallejos et al., 2021).

Another process at the societal level relates to the activation of group membership and other-oriented behavior in the face of a significant challenge (Alonso-Ferres et al., 2020; Fritsche et al., 2008; Schmid and Muldoon, 2015; Wamsler et al., 2023). A longitudinal study (Handschuh et al., 2024) indicated that the sense of social belonging partially mediated the adverse effect of the pandemic on life satisfaction. Specifically, the level of social belonging remained relatively unchanged and was positively associated with individual life satisfaction, which decreased during the pandemic. Simultaneously, empirical studies have often presented the sense of belonging in a limited way, using a single-item measure (e.g., Handschuh et al., 2024) or examining it in the broader context of community resilience (Gilbar et al., 2022; Hall et al., 2023).

**Figure 1:**
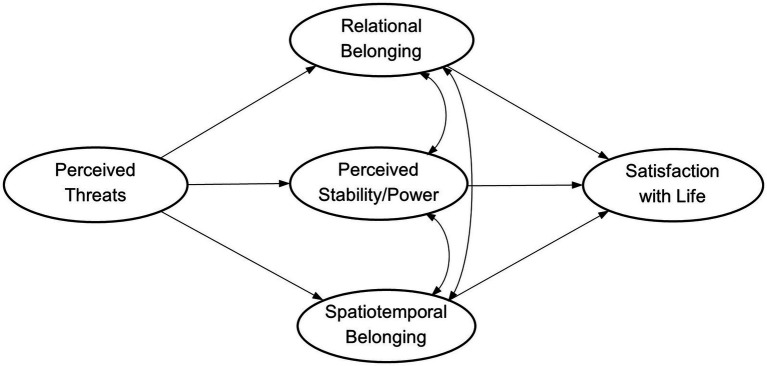
Suggested path model linking perceived global threats to individual satisfaction with life via perceived macro-level characteristics.

To address this limitation, the present study applied a two-component representation of the sense of belonging to the country (Kolesovs, 2021). It included relational and spatiotemporal components, reflecting individual relations with people at the national level and commitment to the country over time. These components present integrative links to social systems and physical environments (Allen et al., 2021; Hagerty et al., 1992), reflecting the basic human need to belong and relate (Baumeister and Leary, 1995; Deci and Ryan, 2000) and perceived continuity of connections to a particular physical context (Kolesovs, 2021). Accordingly, the present study tested a model predicting individual satisfaction with life by the joint factor of perceived epidemiological, ecological, and economic threats via the perceived stability of the country and a complex sense of belonging to it (Figure 1). It should be noted that the perceived macro-level characteristics with potentially protective effects (Gilbar et al., 2022; Hall et al., 2023; Handschuh et al., 2024) were expected to be correlated based on conceptual (e.g., Gilbar et al., 2022) and empirical (Kolesovs, 2019; Kolesovs, 2021) links. In addition, the fully mediated model was tested, regarding perception of a global challenge through the nested macro-system (Bronfenbrenner and Morris, 2006; Pinquart and Silbereisen, 2004).

## 2 Method

The Research Ethics Committee of the Institute of Cardiology and Regenerative Medicine of the University of Latvia approved the study. Data were collected during the first COVID-19 outbreak in Latvia (Centre for Disease Prevention and Control of Latvia, CDPC, 2023). In May and the beginning of June 2020, 317 university students aged 18–48 years (*M* = 23.65, *SD* = 5.98 years) participated in the study. The survey was conducted on paper until June 9, when the Cabinet of Ministers reset strong prevention rules (Cabinet of Ministers of Latvia, 2020). The inventories were distributed by involving a network of social psychologists. Students were invited to participate at the end of lectures and completed the inventory in Latvian without a time limit. Predominantly, the convenience sample consisted of students from social sciences and included 231 (73%) females, 86 (27%) males, and no participants with a declared non-binary identity. Among participants, 189 (60%) were employed, 244 (77%) represented the ethnolinguistic majority group (Latvian speakers), 73 (23%) represented minority groups (non-native Latvian speakers), and 64 (20%) had graduated. The sample size was sufficient for the exploration of the model. Accounting for 160 degrees of freedom within the model, an alternative RMSEA of 0.05, an alpha value of 0.05, and a statistical power of 0.95, the minimal sample size was 175 participants.

The measurements included the Satisfaction with Life Scale (Diener et al., 1985), consisting of five items (*α* = 0.87) and representing a cognitive component of subjective wellbeing. The perceived impact of a joint factor of global threats (Kolesovs et al., 2021) was assessed on a 7-point scale from 1 (no impact) to 7 (maximal impact). In contrast to previous studies (Kolesovs and Ruza, 2019; Kolesovs et al., 2021), participants answered a modified question addressing the current situation: “*To what extent do the factors listed below impact Latvia?*” The list of environmental and economic threats was also extended by adding *climate change* to *epidemics*, *natural disasters*, and *economic crises*.

The perceived stability and power of the country were represented by a three-item semantic differential, which was developed in two steps of a mixed intercultural study (Kolesovs and Kashirsky, 2014). Participants evaluated Latvia using a 7-point scale anchored by the adjectives *powerless–powerful*, *unstable–stable*, and *strong–weak* (a reverse item). Cronbach’s alpha for this subscale in the original study was 0.66. Relational and spatiotemporal components of the sense of belonging (Kolesovs, 2021) presented the sense of belonging to Latvia. Relational belonging was assessed using four items (e.g., “*I feel accepted in Latvia*”). The spatiotemporal commitment subscale asked, “*To what extent do you associate your life with Latvia?*” Participants rated the association level separately for *the recent past*, *present*, *near future*, and *distant future*. For both subscales, respondents used a 7-point scale ranging from 1 (minimally) to 7 (maximally). Cronbach’s alpha scores were 0.82 for the relational component and 0.85 for the spatiotemporal component of the sense.

IBM SPSS Statistics 29.0 (RRID: SCR_016479) was applied for regular statistical tests. Two packages for R Project for Statistical Computing (RRID: SCR_001905) were used for other statistical analyses. The package ‘lavaan’ 0.6–19 (Rosseel, 2012) was applied for structural equation modeling, and ‘semTools’ 0.5–7 (Jorgensen et al., 2022) was used for model invariance testing with robust difference test.

## 3 Results

All scales demonstrated acceptable levels of internal consistency (Table 1), including the perceived power and stability scale, which previously demonstrated reliability of slightly under 0.70 (Kolesovs and Kashirsky, 2014).

**Table 1:** Descriptive statistics and correlations among variables (*N* = 317).

Variables	Items	*α*	*M* (SD) or *n* (%)	1	2	3	4	5
1. Perceived threats	4	0.76	15.53 (5.06)	–				
2. Power and stability	3	0.77	11.39 (3.88)	0.03	–			
3. Relational belonging	4	0.81	17.63 (5.17)	0.19^**^	0.42^***^	–		
4. Spatiotemporal belonging	4	0.86	22.94 (5.29)	0.14^*^	0.30^***^	0.52^***^	–	
5. Satisfaction with life	5	0.85	23.42 (5.47)	−0.01	0.16^**^	0.35^***^	0.34^***^	–
6. Age, years	–	–	23.65 (5.98)	−0.02	−0.11	0.08	0.13^*^	0.15^**^
7. Gender (Female)	–	–	231 (73%)	0.18^**^	−0.06	−0.03	0.01	0.01
8. Employed	–	–	189 (60%)	0.01	0.02	0.04	0.08	0.06
9. Graduated	–	–	64 (20%)	0.05	−0.11	0.02	0.10	0.12^*^
10. Latvian speakers	–	–	244 (77%)	0.09	0.27^***^	0.15^**^	0.15^**^	−0.03

^*^p < 0.05, ^**^p < 0.01, ^***^p < 0.001.

Perceived threats correlated positively with both scales of belonging to Latvia and female gender. Satisfaction with life correlated positively with perceived macro-level variables, age, and graduation level. Age was also positively related to the spatiotemporal component of belonging. In addition, identification with the majority group (Latvian speakers) positively correlated with perceived stability of Latvia and both components of belonging to it.

**Figure 2:**
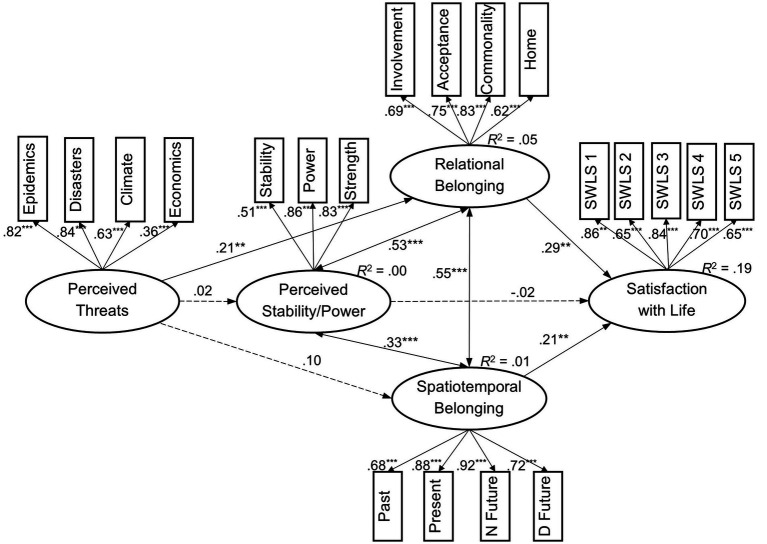
Testing psychological predictors of individual satisfaction with life in the frame of SEM (N Future, Near Future; D Future, Distant Future. Dashed lines present non-significant paths. **p < 0.01, ***p < 0.001).

The structural equation model (Figure 2) demonstrated an acceptable fit: *χ*^2^(161) = 257.59, *p* < 0.001, with the Satorra–Bentler correction factor of 1.15, CFI = 0.96, TLI = 0.95, RMSEA = 0.044 with 90% CI [0.034, 0.053], *p*_(RMSEA < 0.05)_ = 0.877, and SRMR = 0.048. Adding the direct link between perceived threats and satisfaction with life did not significantly alter the model, Δ*χ*^2^(1) = 0.75, *p* = 0.387, ΔCFI = 0.00, ΔRMSEA = 0.000, ΔSRMR = 0.001. Therefore, the suggested model without the direct effect was maintained. The joint factor of perceived threats was not linked to the perceived stability of Latvia or to belonging to the country over time, whereas its link to relational belonging was positive. In turn, perceived macro-level characteristics (except for perceived stability) positively contributed to individual satisfaction with life. Accounting for two sequential significant regressions, a small indirect effect of perceived threats on life satisfaction was found, *β* = 0.06, *p* = 0.013 (*p* = 0.019 after 10^4^ bootstrap draws). The covariances among the relational belonging, spatiotemporal commitment, and perceived stability of Latvia were positive.

**Figure 3:**
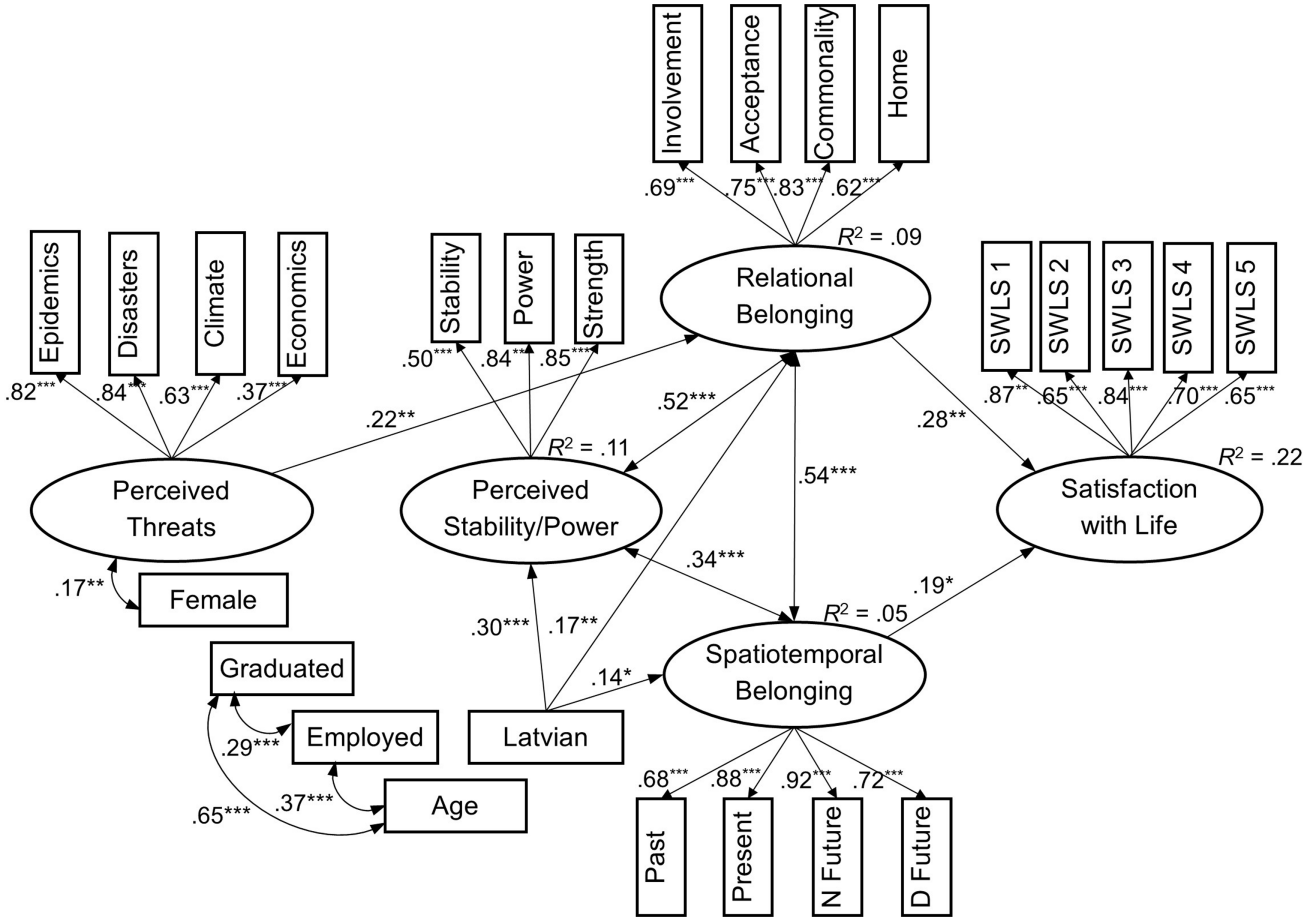
The predictive path model with demographic covariates (N Future, Near Future; D Future, Distant Future. All regressions and covariances were included into analysis, but only significant paths and correlations are presented. *p < 0.05, **p < 0.01, ***p < 0.001).

Testing confirmed strict model invariance (metric, scalar, and residual) regarding students’ gender, Δ*χ*^2^(50) = 40.42, *p* = 0.831, ΔCFI = 0.006, ΔRMSEA = −0.006, working status, Δ*χ*^2^(50) = 51.78, *p* = 0.404, ΔCFI = −0.001, ΔRMSEA = −0.003, majority versus minority status, Δ*χ*^2^(50) = 43.68, *p* = 0.724, ΔCFI = 0.004, ΔRMSEA = −0.005, and graduation, Δ*χ*^2^(50) = 61.48, *p* = 0.128, ΔCFI = −0.004, ΔRMSEA = −0.002. Considering the revealed correlations between psychological and demographic variables, the latter were included in the final model as covariates (Figure 3).

The model with covariates also showed an acceptable fit: *χ*^2^(236) = 372.98, *p* < 0.001, with the Satorra–Bentler correction factor of 1.10, CFI = 0.95, TLI = 0.94, RMSEA = 0.043 with 90% CI [0.035, 0.050], *p*_(RMSEA < 0.05)_ = 0.938, and SRMR = 0.045. Adding covariates did not change conceptual relationships among psychological variables. Being a member of the majority group (Latvian speakers) positively predicted relational belonging, spatiotemporal commitment, and perceived stability of the country.

## 4 Discussion

The results confirmed a joint factor of multiple threats present during the first wave of the COVID-19 pandemic in Latvia. Perceived impact of epidemics was associated with natural disasters, climate change, and economic crises. This grouping concurs with the considerations of Kahn et al. (2022). It also follows the changes observed in perception of health, nature, and economic domains at the very beginning of the COVID-19 pandemic in Latvia (Kolesovs et al., 2021).

The positive link between relational belonging and life satisfaction concurs with the basic role of belonging, relatedness, and social ties in need satisfaction and wellbeing (Allen et al., 2021; Baumeister and Leary, 1995; Deci and Ryan, 2000). The positive effect of perceived global threats on the relational component of the sense of belonging may reflect previously observed activation of social ties as a defensive reaction to threats (Alonso-Ferres et al., 2020; Fritsche et al., 2008; Fung and Carstensen, 2006; Handschuh et al., 2024). An absence of a direct effect of threats on satisfaction with life and a small but positive indirect effect could signify successful coping. However, the positive link was observed under the low prevalence of COVID-19 during the first wave in Latvia (Centre for Disease Prevention and Control of Latvia, CDPC, 2023). Research (e.g., Rogowska et al., 2021) suggests that greater satisfaction with life can be associated with less exposure to COVID-19. As Rogowska et al. (2021) demonstrated, satisfaction with life was higher in countries with relatively lower exposure to the pandemic, and the mean level of satisfaction with life in the current study is close to that of those countries (e.g., Czechia).

The perceived stability of Latvia was independent of joint global threats. On the one hand, students did not perceive Latvia as a highly vulnerable country, which contrasted with findings during the global economic crisis in 2009 and shortly after it, in 2011 (Kolesovs and Kashirsky, 2014). On the other hand, this study was conducted about 3 months after the first registered case in Latvia (Centre for Disease Prevention and Control of Latvia, CDPC, 2023). This temporal interval was probably insufficient to connect the stability of Latvia to the consequences of complex global change, combining health and social issues.

Perceived stability of the country was positively associated with both components of the sense of belonging. These relationships are in line with views of perceived resilience, which combine individual reflections on community resources, including stability and interpersonal relations (e.g., Gilbar et al., 2022). In addition, the link between perceived stability and spatiotemporal commitment to Latvia supports the positive connections between the prospective belonging to it and the perceived situation in the country, its stability, and controllability (Kolesovs, 2019). In turn, a contribution of greater spatiotemporal commitment to life satisfaction confirms the complexity of the sense of national belonging (Kolesovs, 2021) and its positive effects on individual wellbeing under highly challenging conditions (e.g., Handschuh et al., 2024).

The temporal frame significantly limits the generalization of findings to other stages of the pandemic or other global challenges. The opportunity to test the role of perceived macro-contextual factors was the main gain of the study, accounting for the sensitivity of Latvia to global threats (Chzhen, 2016; Gruchoła and Sławek-Czochra, 2021; Kolesovs et al., 2021; Kolesovs and Kashirsky, 2014). However, the dynamics of changes during the pandemic remained unexplored. Unfortunately, other global threats provide opportunities to continue assessing individual reactions to them. The scope of variables was also limited by the frame of a broader project, which was predominantly focused on intentions to emigrate in relation to perceived characteristics of the country. Further studies should address perceived characteristics of lower levels of social systems, affective reactions, a multicomponent perception of wellbeing, socio-economic indicators, and the dynamics of interactions among variables. Moreover, the direction of the links under consideration reflected only one of the possible models, as indicated by previous findings (Handschuh et al., 2024). Alternative models might include, for example, the moderation of perceived links between individual wellbeing and views of the context by perceived situational threats. In addition, the relatively small convenience sample of university students limits the generalization of findings and the power to detect small effects. It should also be noted that educational activities were transformed but not interrupted during the pandemic in Latvia. Therefore, students’ assessment of the situation could be more positive than that of other social groups.

In summary, the present study supported the appearance of health, natural, and economic components in the joint factor of perceived threats during the first wave of the COVID-19 pandemic in Latvia. The relational component of belonging to the country played a central role in linking perceived threats to individual satisfaction with life in a protective manner. Revealed positive effects point to possible strengthening of social ties in the face of a global challenge. Another perceived macro-level characteristic—the country’s stability—was relatively independent of threats and positively linked to the sense of belonging.

## Data Availability

The original contributions presented in the study are included in the article/Supplementary material, further inquiries can be directed to the corresponding author/s.
